# Endothelial biomarkers and platelet reactivity on ticagrelor versus clopidogrel in patients after acute coronary syndrome with and without concomitant type 2 diabetes: a preliminary observational study

**DOI:** 10.1186/s12933-022-01685-4

**Published:** 2022-11-17

**Authors:** Bernadeta Chyrchel, Olga Kruszelnicka, Andrzej Surdacki

**Affiliations:** 1 Second Department of Cardiology, Institute of Cardiology, Faculty of Medicine, Jagiellonian University Medical College, 2 Jakubowskiego Street, 30-688 Cracow, Poland; 2 Department of Cardiology and Cardiovascular Interventions, University Hospital, 2 Jakubowskiego Street, 30-688 Cracow, Poland; 3 Department of Coronary Artery Disease and Heart Failure, Institute of Cardiology, Faculty of Medicine, Jagiellonian University Medical College, 80 Prądnicka Street, 31-202 Cracow, Poland

**Keywords:** Clopidogrel, Endothelial function, Platelets, Ticagrelor, Type 2 diabetes mellitus

## Abstract

**Background:**

Pleiotropic effects have been implicated in clinical benefits of ticagrelor compared to thienopyridine P2Y_12_ antagonists. There are conflicting data regarding effects of ticagrelor vs. thienopyridine P2Y_12_ blockers on endothelial function. Our aim was to compare endothelial biomarkers and their relations with platelet reactivity in real-world patients after acute coronary syndrome (ACS) on maintenance dual antiplatelet therapy (DAPT) with ticagrelor or clopidogrel stratified by diabetes status.

**Methods:**

Biochemical indices of endothelial dysfunction/activation and platelet reactivity by multiple electrode aggregometry were compared in 126 stable post-ACS subjects (mean age: 65 ± 10 years, 92 men and 34 women), including patients with (n = 61) or without (n = 65) coexistent type 2 diabetes (T2DM) on uneventful maintenance DAPT with either ticagrelor (90 mg b.d.) or clopidogrel (75 mg o.d.) in addition to low-dose aspirin. Exclusion criteria included a complicated in-hospital course, symptomatic heart failure, left ventricular ejection fraction < 40% and relevant coexistent diseases except for well-controlled diabetes, mild renal insufficiency or hypertension.

**Results:**

Clinical characteristics were similar in patients on ticagrelor (n = 62) and clopidogrel (n = 64). The adenosine diphosphate-induced platelet aggregation and circulating soluble P-selectin (sP-selectin) were decreased in ticagrelor users irrespective of T2DM status (p < 0.001 and p < 0.01 for platelet reactivity and sP-selectin, respectively). Plasma levels of soluble vascular cell adhesion molecule-1 (sVCAM-1) were lower in T2DM subjects on ticagrelor vs. clopidogrel (758 ± 162 vs. 913 ± 217 µg/L, p < 0.01). In contrast, plasma sVCAM-1 was similar in non-diabetic patients on ticagrelor and clopidogrel (872 ± 203 vs. 821 ± 210 µg/L, p > 0.7). The concentrations of sE-selectin, monocyte chemoattractant protein-1 and asymmetric dimethylarginine did not differ according to the type of P2Y_12_ antagonist regardless of T2DM status. Platelet reactivity was unrelated to any endothelial biomarker in subjects with or without T2DM.

**Conclusions:**

Our preliminary findings may suggest an association of ticagrelor-based maintenance DAPT with favorable endothelial effects compared to clopidogrel users in stable post-ACS patients with T2DM. If proven, this could contribute to more pronounced clinical benefits of ticagrelor in diabetic subjects.

## Background

Dual antiplatelet therapy (DAPT) is a standard of care in patients after acute coronary syndromes (ACS) and/or after coronary stent implantation [1, 2]. In addition to low-dose aspirin, potent P2Y_12_ antagonists, ticagrelor and prasugrel, are preferred over clopidogrel in post-ACS patients, whereas clopidogrel is recommended only if ticagrelor and prasugrel are unavailable, not tolerated or contraindicated, e.g. in patients with high bleeding risk [1, 2].

Beneficial clinical effects of ticagrelor compared to clopidogrel were first demonstrated in the PLATO trial, where ticagrelor was associated with a lower risk of myocardial infarction, cardiovascular (CV) and all-cause death as well as stent thrombosis over a median follow-up of 9 months regardless of ACS type or treatment strategy [3]. Additionally, in the PEGASUS-TIMI 54 trial benefits of prolonged DAPT including low-dose ticagrelor in high-risk patients with prior myocardial infarction 1–3 years earlier were shown [4]. Although relative clinical benefits of ticagrelor in these trials were consistent irrespective of diabetes status [3, 4], absolute risk reduction was greater in patients with coexistent diabetes [5, 6], known to associate with a higher risk of ischemic events via a corollary of mechanisms, including prothrombotic pathways [7, 8]. Moreover, in a GReek AntiPlatElet registry substudy, DAPT with newer P2Y_12_ antagonists, but not clopidogrel, attenuated the negative impact of diabetes on ischemic CV events after ACS [9]. Additionally, net clinical benefits of ticagrelor plus aspirin compared to aspirin alone were reported in participants of the THEMIS-PCI trial with type 2 diabetes (T2DM) and stable coronary artery disease (CAD) with a history of previous percutaneous coronary intervention (PCI) but without prior myocardial infarction or stroke, regardless of diabetes duration or glycated hemoglobin levels [10].

Therefore, keeping in mind a high THEMIS-like population European real-world prevalence [11], the investigation of mechanisms of the beneficial effects of ticagrelor is of relevance. These mechanisms include not only faster, more intense and constant platelet inhibition but also multiple pleiotropic effects, such as anti-inflammatory action, improved coronary flow reserve, downregulation of tissue factor expression, reduced myocardial remodeling and possible improvement of endothelial function, in part linked to enhanced accumulation of endogenous adenosine and vascular P2Y_12_ receptors blockade [12–21].

Nevertheless, both randomized and observational clinical studies focused on comparisons of endothelial effects of ticagrelor and thienopyridine P2Y_12_ antagonists brought conflicting results [14, 15, 22–26]. Importantly, although a recent meta-analysis of randomized controlled studies [27] suggested the ability of ticagrelor to improve brachial flow-mediated dilation (FMD) or reactive hyperemia index (by peripheral arterial tonometry) compared to clopidogrel or prasugrel, the putative effect was mostly driven by reports which had been published only as abstracts.

Importantly, some evidence indirectly suggests that effects of ticagrelor on endothelial function [22, 23, 25, 26] and clinical outcome [6, 28, 29] can be modulated by diabetes status.

In addition, interactions between endothelial function and platelet reactivity appear more complex as P2Y_12_ receptor blockade synergistically potentiates platelet-inhibitory effects of key endothelium-derived mediators, nitric oxide (NO) and prostacyclin (PGI_2_) [30–32].

Our aim was to compare circulating levels of biochemical indices of endothelial dysfunction or activation and their relations with platelet reactivity in real-world post-ACS patients on maintenance DAPT with ticagrelor or clopidogrel stratified by diabetes status.

## Methods

### Patients

We studied hospitalized stable patients aged 30–75 years receiving uneventful maintenance DAPT with either ticagrelor (90 mg b.d.) or clopidogrel (75 mg o.d.) on the background of low-dose aspirin for 1–3 months after PCI for an ACS. The choice of ticagrelor or clopidogrel, initiated previously in the acute phase of ACS, was at the discretion of attending physician.

All the subjects were on angiotensin-converting enzyme inhibitors (ACEI) (or angiotensin receptor blockers [ARB]), high-dose statins, and a proton pump inhibitor (PPI). Exclusion criteria included a complicated in-hospital course, symptomatic heart failure, left ventricular ejection fraction ≤ 40%, congenital heart disease, more than mild valvular heart disease, and estimated glomerular filtration rate (eGFR) < 45 ml/min per 1.73 m^2^ of body surface area according to the Chronic Kidney Disease Epidemiology Collaboration formula. We had also excluded subjects with any relevant coexistent diseases except for well-controlled hypertension or T2DM (diagnosed at least 1 year prior to index hospitalization and treated with hypoglycemic agents), e.g. thyroid dysfunction, neoplastic or inflammatory disease patients, bleeding disorders, patients requiring oral anticoagulation for any indication, as well as those after major surgery or stroke within previous 6 months or with significant abnormalities in routine blood and urine analysis. Additionally, T2DM patients treated with sodium-glucose co-transporter-2 inhibitors or glucagon-like peptide-1 receptor agonists were excluded.

First, we pre-screened 291 consecutive post-ACS patients with regard to exclusion and inclusion criteria, so that similar numbers of diabetic and non-diabetic subjects were selected. Then, we aimed to ensure age- and sex-matching of patients treated with ticagrelor and clopidogrel, i.e. a patient on ticagrelor was matched to his/her counterpart on clopidogrel. Finally, 2 subjects (1 diabetic and 1 non-diabetic woman on ticagrelor) were excluded due to a history of post-discharge switch of P2Y_12_ antagonists, and 126 patients entered the final study.

### Procedure

Blood sampling for the estimation of platelet reactivity and biochemical analyses was performed at routine blood sampling after overnight fast, before the administration of morning doses of drugs, including a maintenance dose of ticagrelor or clopidogrel. Blood for future biochemical assays was drawn from an antecubital vein into ethylenediaminetetraacetic acid-coated tubes, centrifuged, and then plasma was separated and stored in − 80 °C until assayed.

In accordance with the Declaration of Helsinki, the study protocol was approved by the Bioethical Committee of our university (Approval numbers: KBET/277/B/2013 and 1072.6120.143.2019 issued on November 28^th^, 2013 and May 30^th^, 2019, respectively) and informed consent was obtained from the study subjects.

### Platelet reactivity

Platelet aggregatory responses to exogenous adenosine diphosphate (ADP) were assessed in whole blood by multiple electrode aggregometry (MEA) (Multiplate analyzer, Dynabyte, Münich, Germany), based on increased electrical impedance owing to platelet adhesion to the sensor wire surface, as previously described [33, 34]. In brief, a portion of thrombin inhibitor-anticoagulated blood was diluted (1:2) with 0.9% sodium chloride and stirred at 37 °C for 3 min. Then, ADP was added to achieve a final concentration of 6.4 µmol/L and the magnitude of the ADP-induced aggregation was reflected by a rise of impedance in the function of time over a 5-min continuous recording, represented by the area under the curve expressed in arbitrary units (AU) * min [33, 34].

### Biochemical assays

Biochemical analyses included enzyme-linked immunosorbent assays (ELISA) for soluble forms of vascular cell adhesion molecule-1 (sVCAM-1), P-selectin (sP-selectin) and E-selectin (sE-selectin), as well as monocyte chemoattractant protein-1 (MCP-1) (R&D Systems, Minneapolis, MN, USA). According to the manufacturer’s instructions, the lower detection limit was 0.6 µg/L (sVCAM-1), 0.05 µg/L (P-selectin), 0.01 µg/L (sE-selectin) and 1.7 ng/L (MCP-1). For the range of concentrations measured in the study subjects, respective coefficients of variation corresponding to intra-assay (or inter-assay) precision were 2.3% (7.8%), 8.9% (11.9%), 5.1 (7.6%) and 4.9% (4.8%). Additionally, asymmetric dimethylarginine (ADMA) and symmetric dimethylarginine (SDMA) were measured in a subgroup of the study subjects by ELISA (Immundiagnostik AG, Bernsheim, Germany). As previously described [35], the detection limit was 0.04 μmol/L (ADMA) and 0.05 μmol/L (SDMA), while intra-assay (or inter-assay) coefficients of variation averaged 5.8 (7.6%) (ADMA) and 4.8 (7.0%) (SDMA),

### Statistical analysis

Data are shown as means (± SD [standard deviation]), medians [interquartile range] or numbers (percentages). The concordance with a gaussian distribution was assessed by Kolmogorov–Smirnov test and data were natural logarithmically transformed prior to further analyses in case of a non-normal distribution.

Characteristics of patients on ticagrelor and clopidogrel were compared by Student’s t-test or Welch test for continuous variables, and Fisher test for dichotomous characteristics. Homogeneity of variance was checked by Levene’s test.

To estimate the effect of diabetes status on the differences in platelet reactivity, sP-selectin and endothelial biomarkers between ticagrelor and clopidogrel users, a 2-way analysis of variance (ANOVA) was performed followed by Tukey’s test. The analysis of covariance (ANCOVA) was used to control for potential confounding effects of age, body-mass index, glycated hemoglobin and low-density lipoproteins cholesterol. Relations between variables were estimated by Pearson’s (r) correlation coefficients.

A p-value below 0.05 was inferred significant. The Bonferroni correction for multiple comparisons was applied to adjust for possible mutual interrelations of endothelial biomarkers. Our study design had a power of 80% to detect an intergroup difference of about 0.73 SD at an alpha level of 0.05, which corresponds to about 145 µg/L, 125 ng/L and 0.07 µmol/L for the normally distributed endothelial biomarkers VCAM-1, MCP-1 and ADMA, respectively. All analyses were performed by the Statistica 64 (data analysis software system, version 13.3.704.0; TIBCO Software Inc. [2017], Palo Alto, CA, USA).

## Results

Clinical characteristics were similar in our study subjects on ticagrelor and clopidogrel (Table 1).

**Table 1:** Clinical characteristics of patients on DAPT with ticagrelor and clopidogrel stratified by diabetes status

Characteristic	Type 2 diabetes		No diabetes		p^a^
	Ticagrelor n = 30	Clopidogrel n = 31	Ticagrelor n = 32	Clopidogrel n = 33	
Age (years)	66 ± 9	67 ± 10	63 ± 10	63 ± 9	n.s
Men/Women, n (%)	22/8 (73/27)	22/9 (71/29)	24/8 (75/25)	24/9 (73/27)	n.s
Hypertension, n (%)	27 (90)	29 (94)	26 (81)	27 (82)	n.s
Current smoking, n (%)	7 (23)	8 (26)	10 (31)	10 (30)	n.s
Body-mass index (kg/m^2^)	30.5 ± 3.8	29.5 ± 4.0	29.2 ± 2.7	28.4 ± 3.4	n.s
eGFR (mL/min / 1.73 m^2^)	75 ± 17	74 ± 18	80 ± 19	78 ± 17	n.s
LDL cholesterol (mmol/L)	2.0 ± 0.9	1.7 ± 0.8	1.9 ± 0.8	1.9 ± 0.9	n.s
C-reactive protein (mg/L)	2.3 [1.3–3.6]	2.5 [1.3–3.9]	2.0 [1.2–3.1]	2.2 [1.2–3.4]	n.s
Triglycerides (mmol/L)	1.7 ± 0.6	1.9 ± 0.7	1.5 ± 0.7	1.6 ± 0.7	n.s
Multivessel CAD, n (%)	22 (73)	24 (77)	20 (63)	21 (64)	n.s
Left ventricular EF (%)	50 ± 10	50 ± 9	53 ± 10	51 ± 11	n.s
Glycated hemoglobin (%)	7.3 ± 1.0	7.3 ± 0.9	n.d	n.d	n.s
Years from DM diagnosis	5 [3–10]	6 [3–11]	n.a	n.a	n.s
Drugs beyond DAPT, ACEI (or ARB), statin and PPI, n (%)					
Beta-blockers	28 (93)	30 (97)	29 (91)	28 (85)	n.s
Diuretics	12 (40)	10 (32)	9 (28)	10 (30)	n.s
Calcium channel blockers	11 (37)	12 (39)	9 (28)	11 (33)	n.s
Metformin	28 (93)	28 (90)	n.a	n.a	n.s
Sulfonyloureas	7 (23)	7 (23)	n.a	n.a	n.s
Insulin	8 (27)	10 (32)	n.a	n.a	n.s

Data are shown as mean ± SD, median [interquartile range] or n (%)

*ACEI* angiotensin-converting enzyme inhibitors, *ARB* angiotensin receptor blockers, *CAD* coronary artery disease, *DAPT* dual antiplatelet therapy, *DM* diabetes mellitus, *EF* ejection fraction, *eGFR* estimated glomerular filtration rate, *LDL* low-density lipoproteins, *n.a.* not applicable, *n.d.* not determined, *n.s.* not significant, *PPI* proton pump inhibitor,

^a^For diabetic or non-diabetic patients on ticagrelor vs. clopidogrel

**Fig. 1:**
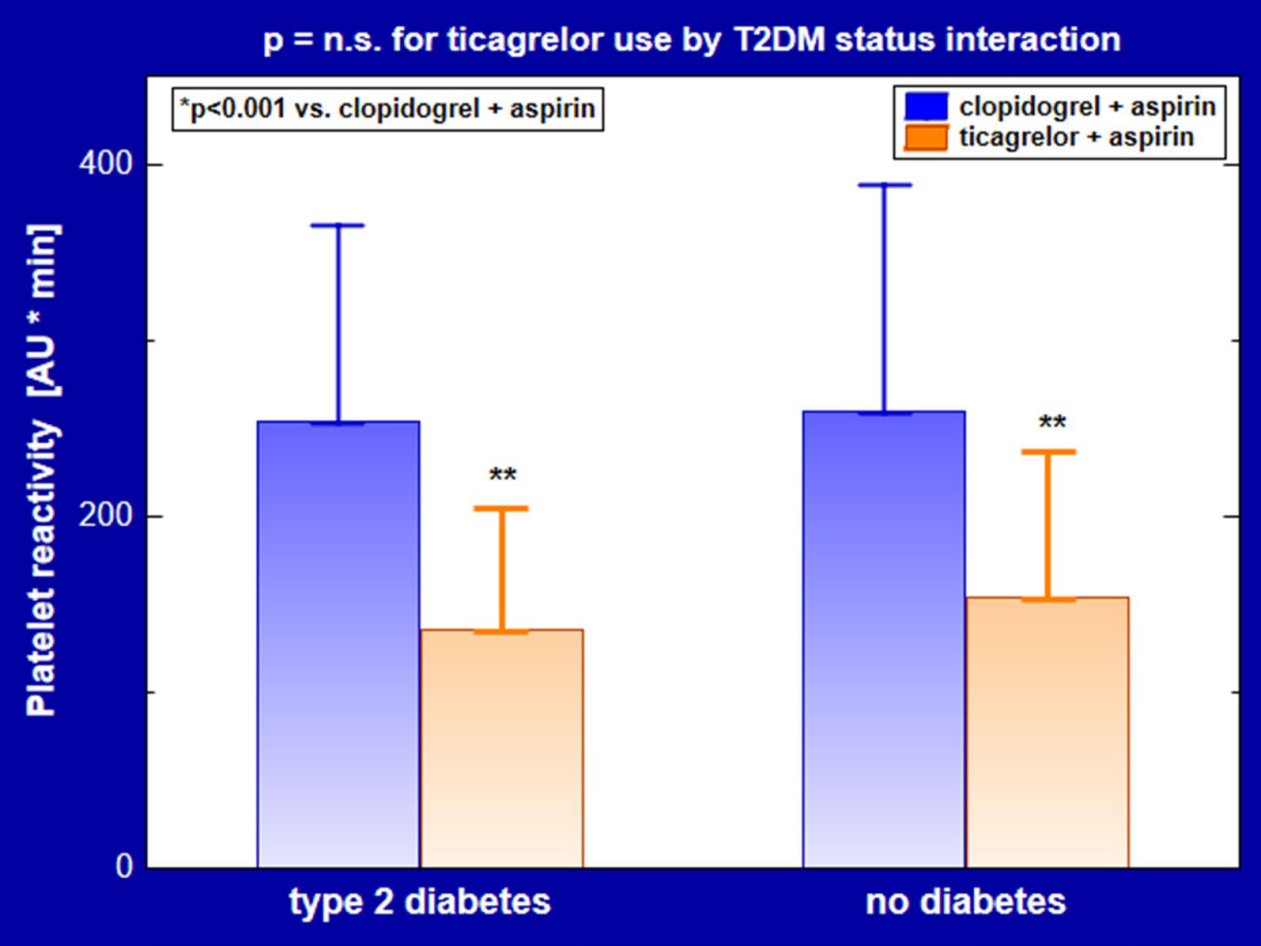
ADP-induced platelet aggregation. Data are shown as mean ± SD

The ADP-induced platelet aggregation was decreased in ticagrelor users compared to their counterparts treated with clopidogrel in patients with (136 ± 69 vs. 254 ± 111 AU * min, p < 0.001) and without T2DM (159 ± 83 vs. 260 ± 128 AU * min, p < 0.001) (Table 2, Fig. 1). Also, plasma sP-selectin levels were decreased on ticagrelor vs. clopidogrel regardless of T2DM status (Table 2).

**Table 2:** Platelet reactivity, sP-selectin and endothelial biomarkers in patients on DAPT with ticagrelor and clopidogrel stratified by diabetes status

Characteristic	Type 2 diabetes		No diabetes		p^a^
	Ticagrelor n = 30	Clopidogrel n = 31	Ticagrelor n = 32	Clopidogrel n = 33	
Platelet aggregability	136 ± 69^**^	254 ± 111	159 ± 83^**^	260 ± 128	n.s
(AU * min)					
sP-selectin (µg/L)	84 [72–106]^*^	114 [83–142]	88 [75–110]^*^	118 [81–145]	n.s.^b^
Endothelial biomarkers					
sVCAM-1 (µg/L)	758 ± 162^*^	913 ± 217	872 ± 203	821 ± 210	< 0.05
sE-selectin (µg/L)	45 [30–76]	48 [32–71]	44 [31–74]	49 [30–77]	n.s.^b^
MCP-1 (ng/L)	451 ± 187	430 ± 165	429 ± 160	449 ± 174	n.s
ADMA (µmol/L)	0.50 ± 0.09	0.49 ± 0.10	0.51 ± 0.10	0.52 ± 0.10	n.s
SDMA (µmol/L)	0.48 ± 0.11	0.49 ± 0.10	0.48 ± 0.10	0.51 ± 0.10	n.s

Data are shown as mean ± SD or median [interquartile range]

*ADMA* asymmetric dimethylarginine, *AU* arbitrary units, *MCP-1* monocyte chemoattractant protein-1, *SDMA* symmetric dimethylarginine, *sE-selectin* soluble E-selectin, *sP-selectin* soluble P-selectin, *sVCAM-1* soluble vascular cell adhesion molecule-1, other abbreviations as in Table 1

^a^For interaction between ticagrelor use and diabetes status by 2-way ANOVA

^b^For log-transformed data

^*^p < 0.01 vs. diabetic patients on clopidogrel (retained significance after Bonferroni correction)

^**^p < 0.001 vs. patients on clopidogrel

**Fig. 2:**
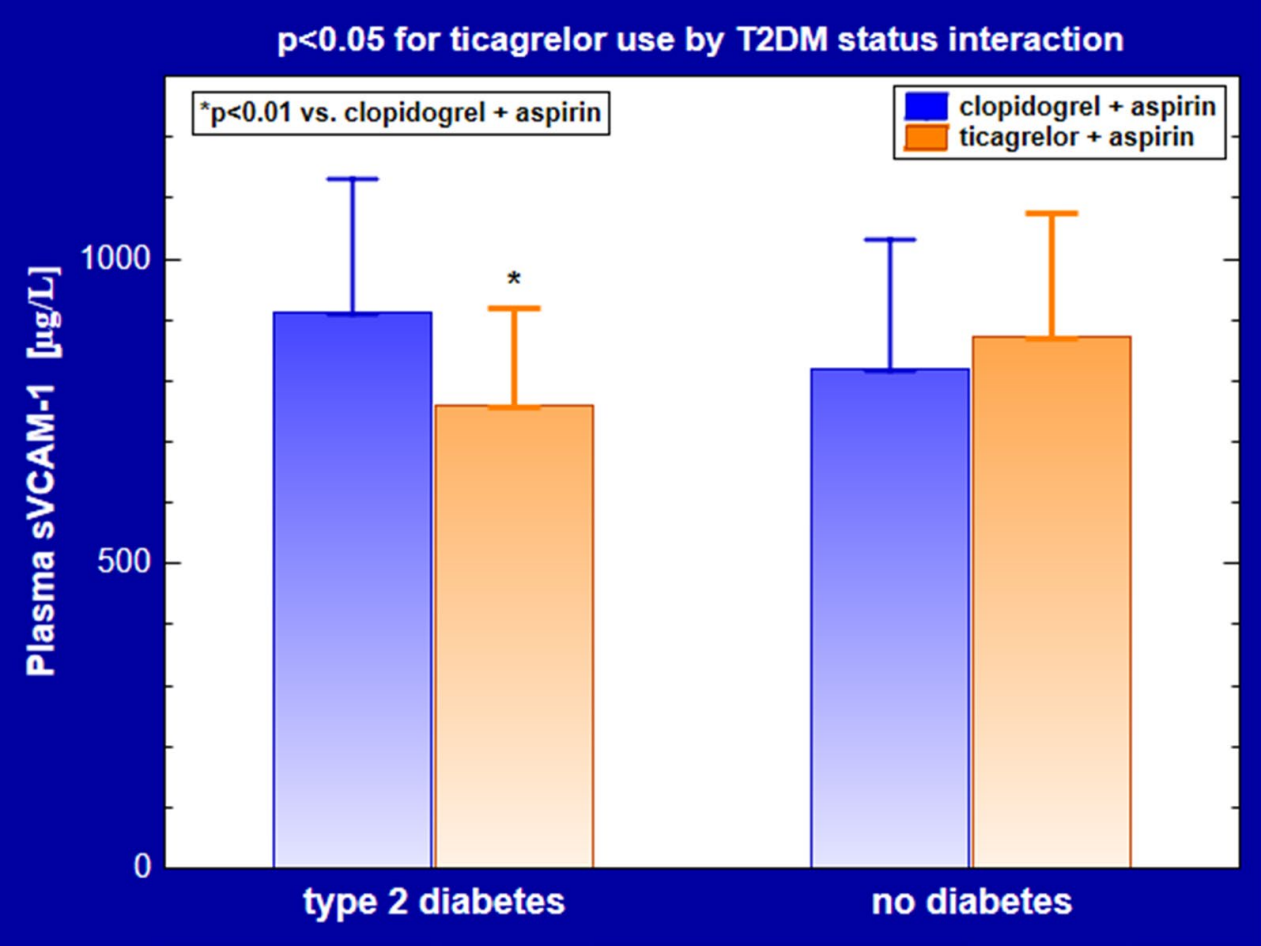
Plasma levels of sVCAM-1. Data are shown as ± mean SD

A 2-way ANOVA revealed a significant interaction between ticagrelor use and diabetes status for plasma sVCAM-1 levels. In patients with T2DM the concentrations of sVCAM-1 were lower in subjects on ticagrelor compared to clopidogrel users (758 ± 162 vs. 913 ± 217 µg/L, p < 0.01) (Table 2; Fig. 2). In contrast, plasma sVCAM-1 was similar in non-diabetic subjects sVCAM-1 n ticagrelor and clopidogrel (872 ± 203 vs. 821 ± 210 µg/L, respectively, p > 0.7) (Table 2; Fig. 2).

The results did not substantially change upon adjustment for potential effects of age, body mass index, glycated hemoglobin and low-density lipoproteins cholesterol by ANCOVA or after exclusion of T2DM subjects taking insulin.

There were no significant differences in the concentrations of sE-selectin, MCP-1, ADMA or SDMA by the type of P2Y_12_ antagonist irrespective of diabetes status. (Table 2). Platelet reactivity was unrelated to any endothelial biomarker in patients with or without T2DM (p > 0.3).

## Discussion

We observed lower circulating sVCAM-1 in post-ACS subjects with T2DM treated with ticagrelor compared to diabetic clopidogrel users, while plasma sVCAM-1 was similar in their non-diabetic counterparts on ticagrelor and clopidogrel. Platelet reactivity and plasma sP-selectin were decreased on ticagrelor vs. clopidogrel regardless of T2DM status. The concentrations of sE-selectin, MCP-1 and ADMA did not differ according to the type of P2Y_12_ antagonist used irrespective of T2DM status. Additionally, platelet aggregability was unrelated to any endothelial biomarker.

To the best of our knowledge, the present study, albeit observational, is the first report focused on comparing endothelial effects of DAPT with ticagrelor vs. clopidogrel in patients with and without T2DM.

### Mechanistic considerations and comparisons with previous studies

Assuming that sVCAM-1, a biochemical index of endothelial activation can be perceived as a surrogate measure of endothelial function, our findings might result from the antiplatelet effect, stronger in patients on ticagrelor vs. clopidogrel. Accordingly, an improvement of endothelial function was previously demonstrated for various P2Y_12_ antagonists, presumably by interactions between platelets, leukocytes and endothelial cells [15, 21, 36, 37]. Additionally, platelet hyperactivity and resistance to DAPT with clopidogrel in T2DM [7, 38], partially dependent on a higher platelet turnover rate, associated with an increased proportion of immature platelets [39], could also favor ticagrelor (administered twice daily) over clopidogrel (dosed once daily). In particular, a gradually emerging subpopulation of juvenile platelets is constantly exposed to an active compound in ticagrelor users, in contrast to clopidogrel whose active metabolite is eliminated from the circulation several hours after prodrug intake, being absent in the systemic blood before the administration of the next dose [21]. Nevertheless, in our hands, a similar degree of platelet inhibition on ticagrelor or clopidogrel was observed in patients with and without T2DM, and on-treatment platelet reactivity was unrelated to any endothelial biomarker, including sVCAM-1 depressed only in diabetic subjects on ticagrelor.

Admittedly, the association of lower sVCAM-1 and ticagrelor use does not imply a cause-and-effect relationship. However, that sVCAM-1 levels were decreased only in T2DM patients on ticagrelor, might suggest a diabetes-specific interaction between endothelial function and ticagrelor use, There is some evidence from randomized crossover studies of CAD patients on DAPT that appears to support, albeit indirectly, this hypothesis [22, 23, 25]. In the CLOTILDIA study, Mangiacapra et al. [22] observed lower platelet reactivity and improved brachial FMD in T2DM patients with stable CAD randomized to ticagrelor vs. high-dose clopidogrel for 2 weeks. In patients with non-ST-elevation ACS and T2DM, Jeong et al. [23] described an improvement of brachial FMD, increased counts of circulating endothelial progenitor cells and decreases in interleukin-6 and tumor necrosis factor-α levels in patients with T2DM randomized to ticagrelor vs. prasugrel for 5 weeks, despite similar platelet reactivity on ticagrelor and prasugrel. In contrast, in stable post-ACS participants of the HI-TECH study (including only 20% of diabetic subjects), Ariotti et al. [25] observed no differences in either peripheral endothelial function or levels of vascular biomarkers (including ADMA, cell adhesion molecules and von Willebrand factor) between subjects on maintenance dose of ticagrelor, prasugrel and clopidogrel for 4 weeks, despite lower platelet reactivity on ticagrelor vs. clopidogrel or prasugrel. Additionally, in a randomized, blinded, parallel study of non-ST-elevation ACS subjects (including 24% of diabetic patients), Schnorbus et al. [26] reported more pronounced platelet inhibition, higher FMD and lower interleukin-6 on DAPT with prasugrel vs. ticagrelor and clopidogrel patients.

Therefore, the improved ability of ticagrelor vs. thienopyridine P2Y_12_ blockers to improve peripheral endothelial function in CAD patients on DAPT may be limited to subjects with coexistent diabetes (mostly T2DM) regardless of whether platelet inhibition on ticagrelor was stronger [22, 25] or not [23, 26] compared to thienopyridines.

As clinical characteristics were similar in our patients on ticagrelor and clopidogrel, intergroup differences are unlikely to account for decreased sVCAM-1 concentrations in our ticagrelor-treated subjects. In contrast to P-/E-selectin-dependent intercellular interactions, responsible for so-called leukocyte rolling along the endothelium, VCAM-1 (binding to very late antigen-4, an a_4_β_1_-integrin expressed on monocytes and lymphocytes) mediates more firm adhesion of leukocytes to endothelial cells and is linked to endothelial activation [40]. Importantly, higher sVCAM-1 (but not soluble forms of E-selectin or intercellular adhesion molecule-1) independently predicted fatal CV events over about 2.7 years in 1246 participants of the Athero*Gene* study with documented angiographic CAD [41]. Accordingly, it can be hypothesized that lower sVCAM-1 might reflect a probable ability of ticagrelor to modulate platelets-monocytes-endothelial interactions [15, 21] in our diabetic subjects with consequent correction of endothelial dysfunction, an antecedent of adverse CV events [42, 43], which could also translate into better long-term prognosis in T2DM subjects on ticagrelor.

Possible mechanisms underlying the association of ticagrelor use with lower sVCAM-1 in our diabetic stable post-ACS subjects remain speculative. Notably, in T2DM ACS patients Jeong et al. [23] reported higher plasma adenosine after ticagrelor loading dose and reduced sVCAM-1 levels after ticagrelor maintenance dose for 5 weeks compared to pre-treatment values. In contrast, Ariotti et al. [25] reported no effects of ticagrelor use on plasma adenosine or any endothelial biomarker (including sVCAM-1) after 4 weeks of drug maintenance dose in predominantly non-diabetic (80%) stable post-ACS subjects. As adenosine induced endothelial NO release [44, 45] and attenuated endothelial activation and VCAM-1 expression in various experimental models [46–49], the ticagrelor-induced enhancement of extracellular adenosine accumulation [16, 17] could account for lower sVCAM-1 levels in our diabetic ticagrelor users.

Second, previously reported differences in blood counts of platelet-derived microvesicles (PMVs) between subjects on DAPT with ticagrelor and clopidogrel might also be linked to intergroup differences in sVCAM-1 levels. Lower counts of PMVs, originating from activated platelets, were described in post-ACS patients on ticagrelor vs. clopidogrel by Gąsecka et al. [50] in a randomized AFFECT EV trial and by our group in an earlier observational study [51]. Additionally, in the former report [50], ticagrelor but not clopidogrel prevented an about twofold increase in the counts of circulating PMVs 6-month after ACS and PMVs counts correlated positively with C-reactive protein levels only in ticagrelor users. Notably, PMVs counts did not correlate with platelet reactivity in either of these studies [50, 51].

PMVs exhibit not only a pro-coagulant [52] but also pro-inflammatory and pro-atherosclerotic activity distantly from the site of platelet activation [53–56], expressing a variety of surface molecules, [53, 57, 58]. Notably, in post-ACS patients, Christersson et al. [59] described not only gradually increasing PMVs counts, but also the association of higher PMVs numbers (but not microvesicles of endothelial or monocytic origin) early after ACS with diabetes and adverse outcome over a 2-year follow-up.

Therefore, lower concentrations of sVCAM-1 in our diabetic patients receiving ticagrelor might reflect the proposed ability of ticagrelor to attenuate excessive PMVs generation, in part independently of the degree of platelet inhibition [50, 51, 60], with consequent lower endothelial activation.

Plasma sP-selectin was significantly lower in both diabetic and non-diabetic patients on ticagrelor vs. clopidogrel. Although P-selectin it is localized not only in platelet alpha-granules but also endothelial Weibel-Palade bodies, circulating sP-selectin mainly reflects platelet activation [61]. Thus, our finding could be ascribed to depressed platelet reactivity in ticagrelor users regardless of T2DM status.

With regard to ADMA, its similar concentrations in our diabetic ticagrelor and clopidogrel users, as opposed to lower sVCAM-1 on ticagrelor, may reflect distinct relationship between ADMA and prognosis in patients with and without T2DM. Accordingly, higher sVCAM-1 predicted adverse clinical outcome in both diabetics and non-diabetics in the population-based Hoorn study [62], while increased ADMA was associated with excessive CV risk in non-diabetic but not diabetic subjects in that [63] and other [64–66] studies.

Nonetheless, regardless of the mechanism involved, possible improvement of endothelial function in diabetic post-ACS patients on ticagrelor might translate into better prognosis.

### Clinical implications: endothelial effects as a possible contributor to clinical benefits of DAPT with ticagrelor in CAD patients with diabetes and other comorbidities

T2DM is associated with worse 1-year outcome in revascularized ACS patients, mainly to excessive risk of ischemic events despite DAPT [7], similar to such comorbidities as chronic kidney disease (CKD) [67], chronic obstructive pulmonary disease (COPD) [68] or peripheral arterial disease [69]. In 2019, Knowles and Warner [70] hypothesized that a synergistic ability of P2Y_12_ blockade and endothelial mediators, PGI_2_ and NO (and their intraplatelet second messengers, cyclic adenosine 3’,5’-monophosphate (cAMP) and cyclic guanosine 3’,5’-monophosphate (cGMP), respectively) to inhibit platelet aggregation [30, 32, 71] might be of particular clinical relevance in post-ACS subjects with the above mentioned comorbidities, sharing widespread endothelial dysfunction and chronic inflammatory activation. Importantly, endothelial dysfunction extends also to peripheral and pulmonary microvessels where platelets undergo continuous and prolonged exposure to endothelial mediators due to a close proximity to numerous endothelial cells and slow blood flow velocity compared to large arteries [70].

Therefore, depressed generation of NO and PGI_2_ might also limit the efficacy of even potent P2Y_12_ antagonists despite DAPT in post-ACS patients with coexistent comorbidities, including T2DM, keeping in mind a three-way synergistic interaction between P2Y_12_ blockade and endothelial mediators, NO and PGI_2_, for adequate platelet inhibition [30–32, 71]. Moreover, a possible ability of ticagrelor to both inhibit P2Y_12_ receptors and improve endothelial function in T2DM might at least partially restore the synergistic antiplatelet effect with a subsequent reduction of ischemic CV events, more pronounced in CAD with comorbidities, including T2DM.

In agreement with the proposed hypothesis, in the ISAR-REACT 5 trial, a better ability of DAPT with prasugrel than ticagrelor [72] to reduce the risk of ischemic CV events or definite stent thrombosis was limited to non-diabetic ACS patients undergoing PCI [28], whereas in diabetic subjects an insignificant opposite tendency in favor of ticagrelor was observed [28].

Additionally, the hypothetical diabetes-specific endothelial effects of ticagrelor might have also contributed to stronger reductions of the risk of CV death and coronary death in diabetic vs. non-diabetic participants of the PEGASUS-TIMI 54 trial with a history of myocardial infarction 1–3 years earlier receiving ticagrelor (60 or 90 mg b.d.) with aspirin vs. aspirin alone [6], as well as to a lower risk of ischemic CV events with ticagrelor (60 mg b.d., most prevalent dose) as an addition to aspirin in T2DM subjects with stable CAD after prior PCI without previous myocardial infarction or stroke in the THEMIS-PCI trial [10, 73].

Also, in the NATHAN-NEVER study, a randomized, parallel trial including stable CAD patients with coexistent COPD, beneficial effects for 1-month DAPT with ticagrelor vs. clopidogrel with regard to a set of parameters related to endothelial function were reported, including lower circulating epidermal growth factor levels, reduced apoptosis rate and enhanced NO generation in cultured endothelial cells incubated with patients’ serum, as well as lower reactive oxygen species generation and increased *SIRT1* and *HES1* mRNA levels in peripheral leukocytes [24, 74, 75].

### Future perspectives: can endothelial effects contribute to clinical benefits of ticagrelor monotherapy in CAD patients with diabetes ?

Notably, Tam et al. [76] have recently described improved FMD in patients with non-significant CAD (including only 25% of diabetics) randomized to low-dose ticagrelor (60 mg b.d.) for 12 weeks compared to low-dose aspirin despite comparable platelet activation markers. Hence, endothelial effects of ticagrelor can also extend to ticagrelor monotherapy. Ticagrelor monotherapy, following shortened DAPT, was recently proposed as an emerging strategy in patients after coronary stent implantation both after ACS and in chronic coronary syndromes [29, 77, 78].

In the GLOBAL LEADERS trial in the second year after angioplasty, ticagrelor monotherapy (90 mg b.d.) was more effective than aspirin monotherapy in lowering the risk of ischemic CV events in those with coexistent both diabetes and CKD [78, 79]. Thus, since an improvement of FMD on ticagrelor vs. aspirin was reported by Tam et al. [76] at a ticagrelor dose of 60 mg b.d., endothelial effects could possibly contribute to a superiority of ticagrelor monotherapy at a dose of 90 mg o.d. over aspirin alone for ischemic risk in some subgroups of CAD patients with comorbidities who had angioplasty 12–24 months earlier [79].

In the TWILIGHT trial, there was a marginal (p = 0.05) diabetes by treatment interaction, driven mostly by a lower risk of myocardial infarction at 15 months after high-risk PCI in diabetic patients on ticagrelor monotherapy (90 mg b.d.) vs. ticagrelor plus aspirin after 3-month DAPT with ticagrelor [29, 77]. This unexpected and counterintuitive finding is consistent with previous observations of only limited additional platelet inhibition by aspirin in the presence of strong P2Y_12_ blockade by ticagrelor or prasugrel active metabolite [70, 80, 81]. Moreover, a considerable aspirin-induced inhibition of non-platelet cyclooxygenase with consequent reduction of PGI_2_ generation was reported already at low aspirin doses [82]. Therefore, even low-dose aspirin might impair the synergism between intraplatelet cAMP and cGMP [71], second messengers of PGI_2_ and NO, respectively, responsible for continuous platelet inhibition [70].

It is intriguing why the above described aspirin-induced detrimental mechanistic effect [70, 82] could adversely affect prognosis exclusively in ticagrelor users with coexistent T2DM in the TWILIGHT trial [29, 77]. Nonetheless, it could be speculated that this finding might reflect a more pronounced dependence of clinical benefits of ticagrelor on an intact three-way synergism between P2Y_12_ blockade and endothelial mediators, PGI_2_ and NO [30, 32, 70, 71] in T2DM, owing to platelet hyperactivity in diabetic patients [7, 38, 39]. Additionally, this concept appears consistent with the ticagrelor-dependent enhanced accumulation of adenosine [16, 17] that increases endothelial NO release [44,45] and intraplatelet formation of cAMP [83], a PGI_2_ second messenger.

## Limitations of the study

First, an observational, not randomized, study design and a low number of patients involved pose considerable limitations. Nevertheless, we applied a wide set of exclusion criteria, recruiting a relatively homogenous group of stable post-ACS subjects receiving uneventful maintenance DAPT without heart failure or coexistent diseases except for well-controlled diabetes, hypertension or mild eGFR reduction. Moreover, clinical characteristics were similar in balanced subgroups of patients on ticagrelor and clopidogrel and potential effects of selected confounders were excluded by ANCOVA. Additionally, ACEI (or ARB), a high-dose statin and PPI were used by all the study patients, while the proportions of subjects on beta-blockers, calcium channel blockers or diuretics were similar by ticagrelor use or diabetes status. Although we had previously reported lower sVCAM-1 levels in stable CAD patients with T2DM on metformin [35], the prevalence of metformin use was comparable in our T2DM subjects receiving ticagrelor and clopidogrel. Second, we have analyzed only biomarkers linked to endothelial dysfunction or activation, with no assessment of functional indices such as FMD or reactive hyperemia index by arterial tonometry. However, our in-house estimation has detected a relatively large individual variability of FMD and the need for long procedure in case of peripheral tonometry, which would limit its feasibility in clinical conditions. Third, we assessed platelet reactivity as ADP-induced platelet aggregation by MEA instead of a specific vasodilator-stimulated phosphoprotein phosphorylation (VASP-P) assay focused on the P2Y_12_-dependent effect. Nevertheless, platelet reactivity by MEA was a better predictor of stent thrombosis than the VASP-P assay in the PEGASUS-PCI study [84].

## Conclusions

Our preliminary findings may suggest an association of ticagrelor-based maintenance DAPT with favorable endothelial effects compared to clopidogrel users in stable post-ACS patients. If proven, this could contribute to more pronounced clinical benefits of ticagrelor in diabetic subjects.

## Data Availability

The dataset supporting the principal conclusions of the current study is available from the corresponding author on reasonable request.

## Abbreviations

ACEI: Angiotensin-converting enzyme inhibitors
ACS: Acute coronary syndrome
ADMA: Asymmetric dimethylarginine
ADP: Adenosine diphosphate
ANCOVA: Analysis of covariance
ANOVA: Analysis of variance
ARB: Angiotensin receptor blockers
AU: Arbitrary units
CAD: Coronary artery disease
cAMP: Cyclic adenosine 3’,5’-monophosphate
cGMP: Cyclic guanosine 3’,5’-monophosphate
CKD: Chronic kidney disease
COPD: Chronic obstructive pulmonary disease
CV: Cardiovascular
DAPT: Dual antiplatelet therapy
EF: Ejection fraction
eGFR: Estimated glomerular filtration rate
FMD: Flow-mediate dilation
LDL: Low-density lipoproteins
MCP-1: Monocyte chemoattractant protein-1
MEA: Multiple electrode aggregometry
NO: Nitric oxide
PPI: Proton pump inhibitor
PCI: Percutaneous coronary intervention
PGI_2_: Prostacyclin
PMVs: Platelet-derived microvesicles
SD: Standard deviation
SDMA: Symmetric dimethylarginine
sE-selectin: Soluble E-selectin
sP-selectin: Soluble P-selectin
sVCAM-1: Soluble vascular cell adhesion molecule-1
T2DM: Type 2 diabetes mellitus
VASP-P: Vasodilator-stimulated phosphoprotein phosphorylation
